# CA15-3 is a useful serum tumor marker for diagnostic integration of hybrid positron emission tomography with integrated computed tomography during follow-up of breast cancer patients

**DOI:** 10.1186/1471-2407-14-356

**Published:** 2014-05-21

**Authors:** Mariarosaria Incoronato, Peppino Mirabelli, Onofrio Catalano, Marco Aiello, Chiara Parente, Andrea Soricelli, Emanuele Nicolai

**Affiliations:** 1 IRCCS Fondazione SDN, Via E. Gianturco n°113, Naples 80143, Italy; 2 Università degli studi di Napoli Parthenope, Via Ammiraglio Ferdinando Acton n°38, Naples 80133, Italy

**Keywords:** Breast cancer, Serum biomarkers, CA15-3, Positron emission tomography, Computed tomography

## Abstract

**Background:**

The aim of this study was to evaluate the value of CA15-3 for the diagnostic integration of molecular imaging findings performed with hybrid positron emission tomography and computed tomography (PETCT) technology.

**Methods:**

We retrospectively selected 45 patients with a median age of 60 years (range 39–85 years) and a previous history of breast cancer (BC) who had already been treated with surgery and other treatments. Three measurements of CA15-3 were collected within 1 year before PETCT examination, at 6–9 months 3–6 months and 0–3 months before PETCT. The prolonged clinical outcome or imaging follow-up was used to define disease relapse. An increase in tumor marker value was compared with PETCT findings and disease relapse. Sensitivity and specificity for both tests were calculated with respect to clinical outcome.

**Results:**

Disease relapse was detected in 16 out of 45 BC patients. CA15-3 and PETCT showed 75% sensitivity with a specificity percentage of 76% for CA15-3 and 79% for PETCT. Serum CA15-3 expression levels were significantly higher in BC patients with multiple metastatic sites with hepatic involvement. Analysis of serial CA15-3 serum levels showed an increase in CA15-3 3–6 months before PETCT could identify BC patients at risk for relapse (AUC = 0.81). Moreover, patients receiving anti-hormonal or chemotherapy medications with negative PETCT and positive CA15-3 relapsed after a median time of 158 days compared to patients who were negative for both tests and who were free from disease for at least 1 year.

**Conclusions:**

Our results showed that serial increases in CA15-3 can be used to predict positive PETCT results in BC patients during follow-up. Increased levels of CA15-3 may be considered an early warning sign in patients needing accurate molecular imaging investigations, as they are at higher risk of recurrence. In cases of elevated levels, multiple lesions or liver involvement may exist. Also, patients receiving chemotherapeutic or anti-hormonal treatment who have negative PETCT scans and increased CA15-3 serum levels should be considered at risk for relapse, because the CA15-3-linked biochemical signal of the presence of a tumor can predict positive metabolic imaging.

## Background

Breast cancer (BC) is by far the most frequent type of cancer in women worldwide, but it is associated with relatively lower mortality rates, as it ranks fifth in cancer-related deaths overall [1]. These data mostly reflect improvements in the treatment of BC recurrences and metastases due to the availability of new drugs and biological therapies [1]. The efficacy of these medications is greatly improved if recurrent or metastatic disease is detected early [2]. Thus, during recent years, the introduction of 2-deoxy-2-[^18^ F] fluoro-d-glucose (^18^FDG) positron emission tomography (PET) with integrated computed tomography (CT), known as PETCT, has become a helpful tool for this purpose [3]. Indeed, PETCT is able to detect recurrences at very early stages of development due to the abnormal uptake of ^18^FDG by neoplastic cells before the onset of morphologic changes that are detectable with conventional imaging [4,5]. Whole-body PETCT is a very useful diagnostic technology, especially when BC recurrence is suspected in asymptomatic patients with elevated tumor marker levels and negative conventional imaging results [6-8]. In this case, the early detection of disease relapse greatly improves the chances for successful treatment. However, despite this important feature, an intense debate remains among scientific organizations about the value of tumor markers in predicting clinical or radiological findings of disease relapse [9]. To date, only the European Group on Tumor Markers (EGTM) suggests that an increase in serum CA15-3 often predicts clinical or radiological signs of disease recurrence in BC patients, with an estimated lead time of 2–9 months [10-12].

In this study, we performed a retrospective analysis of the medical records of 45 BC patients with suspicion of recurrence who underwent both PETCT scans and serial measurements of CA15-3 to asses if: i) a serial increase in CA15-3 was optimal for recommending addressing PETCT examination of BC patients during follow-up; ii) CA15-3 serum levels could be correlated with PETCT findings in terms of number of detectable FDG positive lesions; iii) elevated CA15-3 serum levels are also predictive of disease relapse in patients who at the end of their therapeutic treatment have a negative PETCT result and are positive for CA15-3.

## Materials and methods

### Patient population

A review of the biochemistry and molecular imaging databases was performed at the SDN Institute to select BC patients who underwent both PETCT scans and serial CA15-3 serum level measurement for suspicion of recurrence as required for their clinical care protocol between January 2011 and December 2012. Based on these criteria, we selected one male and 44 female patients with a median age of 60 years (range 39–85 years) who underwent clinical follow-up in the routine oncology setting. Sixteen out of 45 BC patients experienced a BC relapse, 11/16 patients underwent chemotherapy, 1/16 underwent chemotherapy with Herceptin®, 2/16 underwent surgery plus chemotherapy, and 2/16 started or continued hormonal therapy (Tamoxifen or inhibitors of aromatase). Among the patients without disease relapse, 6/29 continued hormonal therapy, and the remaining patients were followed up after surgical or chemotherapeutic intervention. All patients without signs of disease relapse remained free from disease for at least 1 year after the PETCT scans and CA15-3 measurements that were performed in this study.

Informed consent for PETCT examination was obtained from all patients. This study was approved by the Ethics Committee of IRCCS Fondazione SDN (Naples, Italy). Because this was a retrospective study and all procedures had already been performed for clinical purposes, our Institutional Review Board (Ethics Committee of IRCCS Fondazione SDN) did not require further patient approval or informed consent for the review of patient files or images.

### ^18^FDG PETCT Imaging

PETCT scans were performed with a hybrid system (Gemini TF Philips Healthcare, Best, Netherlands). Before the PETCT scan, each patient fasted for at least 6 h, and the blood glucose level (glucose level below 7.78 mmol/L) was measured prior to injection of 4–5 MBq/kg of ^18^FDG. One hour after injection, the PETCT scan was acquired in 3D and was thus fully reconstructed with the line-of-response based row-action maximum-likelihood algorithm (LOR RAMLA). In particular, acquired images were processed to obtain trans-axial, sagittal, and coronal views. The PETCT system obtains precise anatomic localization of ^18^FDG positive lesions, and CT-based attenuation correction ensures an accurate quantitative measurement of the standardized uptake value (SUV) of lesions. During the acquisition, the patient was in a supine position with both arms brought together above the head. All acquired images were reviewed with a Xeleris workstation (General Electric Heathcare, Milwaukee, WI, USA) and interpreted by an experienced nuclear medicine physician and a radiologist to obtain a final diagnosis by consensus. Regions showing focally prominent FDG-PET uptake compared with surrounding tissues and not related to normal physiologic uptake were considered positive for malignancy. The Xeleris workstation produces SUVs that are calculated as the ratio of the tissue radioactivity concentration measured with PET and the injected dose divided by the patient’s weight. In the reviewing procedure, physicians assigned the maximum SUV to each lesion within the region of interest (SUVmax). Furthermore, CT provides accurate information about the anatomical localization of such regions. For BC patients with disease relapse, a volumetric characterization of lesion burden was made [13], considering a metabolic tumor volume (MTV) with a threshold of 40% of the maximum signal intensity (MTV40). Therefore, a voxel with an SUV between 40% of SUVmax and SUVmax was considered to be part of the lesion.

### Measurement of CA15-3

The CA15-3 serology test was performed at the laboratory of clinical biochemistry at the SDN Institute in accordance with the manufacturer’s protocols and reference intervals. Specifically, all serum tumor markers were measured on an ADVIA Centaur® XP automated Immunoassay System (SIEMENS Healthcare Diagnostics Inc., Tarrytown, NY, USA). The threshold value provided by the supplying company for CA15-3 was 25 UI/mL. For each patient, three serial serum CA15-3 determinations performed at 6–9 months (270 ± 102 days), 3–6 months (139 ± 79 days), and 0–3 months (20 ± 30 days) before PETCT were recovered from the SDN archives.

### Statistical data analysis

All statistical analyses were performed using GraphPad Prism version 4.0 (GraphPad Software, La Jolla, CA, USA). Continuous data were presented as the mean ± standard deviation or median ± interquartile range. Statistical significance was evaluated with the Mann–Whitney U-Test for unpaired data or the Student’s t test as appropriate. Area Under the Curve (AUC) was calculated with Receiving Operating Characteristic (ROC) analysis, to study the variation in CA15-3 to better predict a positive PETCT result at different time points.

## Results and discussion

The daily clinical experiences in monitoring BC patients after primary therapy demonstrate that any significant increase in tumor markers, even in the absence of other clinical or instrumental signs of cancer, justifies PETCT examination [14,15]. Nevertheless, the usefulness of biomarkers in monitoring BC patients after primary therapy is still the subject of intense debate in the scientific literature [16]. In particular, the current guidelines of the American Society of Clinical Oncology do not recommend measurement of serum biomarkers such as CarcinoEmbryonic Antigen (CEA) and CA15-3 for monitoring BC patients during follow-up [17]. Conversely, the EGTM, in agreement with the National Academy of Clinical Biochemistry guidelines and the European Association for Nuclear Medicine, suggests that increasing levels of serum tumor markers may often precede clinical or radiological signs of disease recurrence [18,19]. In addition to these recommendations, a growing number of scientific papers have re-evaluated the value of increased CA15-3 serum levels for early detection of recurrence, showing that serum determination of CA15-3 improves the diagnostic accuracy of PETCT [20-23].

Given these reports, we decided to perform a retrospective study of a group of 45 BC patients who were followed up in our institution with PETCT scans and measurement of CA15-3 serum levels according to their clinical care protocol. Disease relapse was documented by prolonged clinical or radiological follow-up in 16 out of 45 patients (35%). In relapsing patients, the CA15-3 levels were ≥25 UI/mL in 12 out of 16 cases (75% sensitivity), whereas in disease-free patients, CA15-3 levels were <25 UI/mL in 22 out of 29 cases (76% specificity). The CA15-3 positive and negative predictive values were 63% and 84%, respectively. PETCT was able to detect recurrence or metastatic disease in 12 out of 16 patients (75% specificity), whereas in 23 out of 29 (79%) non-relapsing BC cases, PETCT did not show pathological SUV sites. The positive and negative predictive values of PETCT were 67% and 85%, respectively.

For diagnostic integration of the two methods, we correlated the CA15-3 serum levels with PETCT findings and found a significant increase in the median CA15-3 serum levels (p-value <0.05) in patients with multiple metastatic sites (8 out of 12 patients with positive PETCT) compared to those with a single metastatic site (4 out of 12 patients with positive PETCT). Specifically, in relapsing BC patients with multiple metastatic sites, the median value of CA15-3 was 49.1 UI/mL (range 8.2–298.4 UI/mL), whereas in those with a single metastatic site, the median value was 17.4 UI/mL (range 5.8–44.2 UI/mL).These data showed that, according to previous studies, elevated levels of CA15-3 are predictive of disease dissemination rather than local recurrence [24-26]. In addition, the highest serum levels of CA15-3 (≥120 UI/mL) were found in patients with hepatic involvement in agreement with Nicolini and colleagues [27].

**Figure 1:**
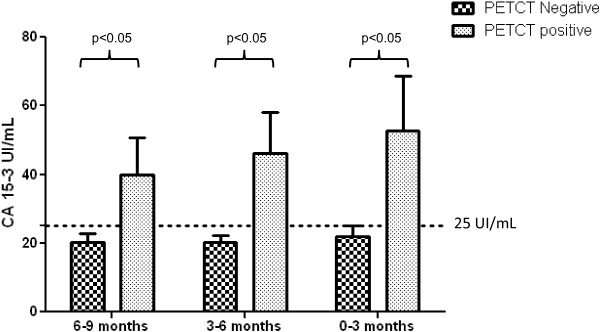
**Correlation between the trend in serial CA15-3 measurements and PETCT findings.** The bar plot shows the mean CA15-3 serum levels at 0–3, 3–6, and 6–9 months prior to PETCT. Dotted line indicates CA15-3 cut-off (25 UI/mL). The P-value was determined with an unpaired t-test.

**Figure 2:**
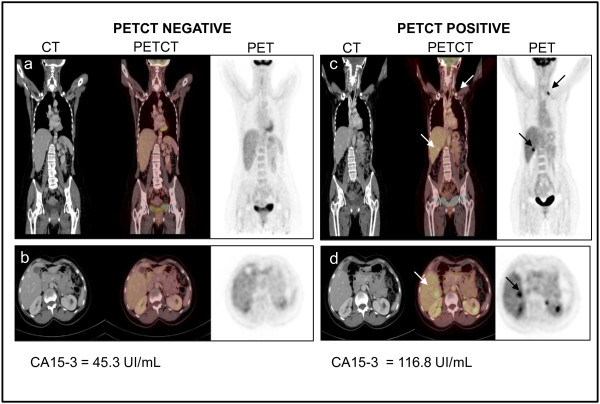
**Right side: example of a patient with negative PETCT findings (a: coronal view; b: transaxial view) and CA15-3 levels of 45.3 UI/mL; left side: the same patient 6 months later with positive PETCT findings (c: coronal view; d: transaxial view) as well as CA15-3 levels of 116.8 UI/mL.** Arrows indicate the metastatic sites.

Based on these findings, we asked if CA15-3 provides earlier detection of a relapse in comparison to PETCT. To test this idea according to other studies [22,26], for each patient, we collected three serial serum CA15-3 measurements from our internal archives. As shown in Figure 1, the serial measurements were performed at 6–9 months (270 ± 102 days), 3–6 months (139 ± 79 days), and 0–3 months (20 ± 30 days) before the PETCT scan. In patients with a positive PETCT scan, the mean CA15-3 value 6–9 months before the PETCT scan was 40.01 ± 42.40 UI/mL (median 23.3 UI/mL, range 6.2–159 UI/mL), 3–6 months before the PETCT scan was 46.17 ± 42.97 UI/mL (median 29.20 UI/mL, range 8–155 UI/mL), and 0–3 months before the PETCT scan was 52.64 ± 68.33 UI/mL (median 31.9 UI/mL, range 5.8–298.4 UI/mL). As expected, in patients with a negative PETCT scan, the mean CA15-3 values 6–9 months, 3–6 months, and 0–3 months before the PETCT scan were <25 UI/mL. In PETCT positive cases the increase in the percentage of CA15-3 between the time spans of 3–6 and 6–9 months was 15%, whereas that between 0–3 and 3–6 months was 14%. Moreover, using ROC curve analysis, we found that the AUC value for CA15-3 was 0.81 for both 0–3 and 3–6 months before the PETCT scan and 0.77 6–9 months before the scan. According to Evangelista et al. [22], these results indicate that an increase in CA15-3 3–6 months (139 ± 79 days) before PETCT could already identify BC patients at risk for relapse, and thus, the biochemical signal of the presence of a tumor could predict positive metabolic imaging of disease. This conclusion is interesting especially in BC patients who undergo PETCT examination while receiving anti-hormonal or chemotherapy medications. In fact, these medications reduce the tumor avidity for FDG and consequently, the sensitivity of PETCT [22]. In our series, 10 BC patients were receiving therapeutic treatment, and PETCT and CA15-3 serum levels were obtained every 6 months. In four of those 10 patients, PETCT was negative and CA15-3 serum levels were higher than 25 UI/mL (45.3, 84.6, 27.8, and 26.2 UI/mL). Nevertheless, they relapsed after a median time of 158 days and showed an increase in the CA15-3 serum levels (116.8, 96.4, 44.2, and 30 UI/mL) and a positive PETCT scan. Conversely, the control group, which was composed of the remaining patients (n = 6) who had a negative PETCT scan and low CA15-3 levels, was free from disease even after 1 year of follow-up. Figure 2 shows an example patient at the end of chemotherapy treatment with a negative PETCT result and a positive CA15-3 value (45.3 UI/mL). This patient relapsed after 6 months and showed positive PETCT findings and CA15-3 levels that had increased 2.5-fold (116 UI/mL). These results show for the first time the usefulness of CA15-3 for identifying patients with false negative PETCT scans that require a stringent follow-up due to a risk for developing disease relapse.

## Conclusions

Our study, although performed with a small case series, shows that a serial increase in CA15-3 can predict positive PETCT results and detect the presence of residual disease in patients during therapeutic treatment who show negative PETCT results. The diagnostic integration of CA15-3 with PETCT could be a useful strategy for optimizing clinical protocols for early detection of disease relapse. Indeed, the monitoring of CA15-3 may be useful for correct timing of performing PETCT for early detection of cancer lesions and initiation of appropriate therapeutic intervention. Further studies are necessary to identify additional serum biomarkers that can be integrated with modern molecular imaging instruments. Indeed, CA15-3 is not useful in cases of local regional recurrences or for screening purposes. Innovative biomarkers such as microRNAs and circulating tumor DNA are promising, however, they need to be validated and translated into clinical practice.

## Competing interests

No grant funding was received for this study, and there are no competing interests to declare.

## Authors’ contributions

MI and PM conceived the study, performed statistical data analysis, and wrote the manuscript; MI performed serum marker analysis; OC and EN performed the PETCT clinical evaluation; MA performed the data processing; CP revised the statistical data analysis; AS, OC, and EN reviewed and revised the manuscript for important intellectual content. All authors gave final approval of the version to be published.

## Pre-publication history

The pre-publication history for this paper can be accessed here:

http://www.biomedcentral.com/1471-2407/14/356/prepub

## Aknowledgements

This work was supported by Italian Ministry of Health (Current Research Program RC-2012). We thank Manjusha Sala, Assistant Managing Editor, who provided medical writing services on behalf of BioScience Writers LLC.

## References

[B1] Bray F Ren JS Masuyer E Ferlay J Global estimates of cancer prevalence for 27 sites in the adult population in 2008 Int J Cancer 2013 132 1133 1145 10.1002/ijc.27711 22752881

[B2] Cardoso F Harbeck N Fallowfield L Kyriakides S Senkus E on behalf of the ESMO Guidelines Working Group Locally recurrent or metastatic breast cancer: ESMO Clinical Practice Guidelines for diagnosis, treatment and follow-up Ann. Oncol 2012 23 Suppl 7 vii11 vii19 22997442 10.1093/annonc/mds232

[B3] Gil-Rendo A Martínez-Regueira F Zornoza G García-Velloso MJ Beorlegui C Rodriguez-Spiteri N Association between [18 F] fluorodeoxyglucose uptake and prognostic parameters in breast cancer Br J Surg 2009 96 166 170 10.1002/bjs.6459 19160365

[B4] Groheux D Giacchetti S Moretti JL Porcher R Espié M Lehmann-Che J de Roquancourt A Hamy AS Cuvier C Vercellino L Hindié E Correlation of high 18 F-FDG uptake to clinical, pathological and biological prognostic factors in breast cancer Eur J Nucl Med Mol Imaging 2011 38 426 435 10.1007/s00259-010-1640-9 21057787

[B5] Wang Y Zhang C Liu J Huang G Is 18 F-FDG PET accurate to predict neoadjuvant therapy response in breast cancer? A meta-analysis Breast Cancer Res Treat 2012 131 357 369 10.1007/s10549-011-1780-z 21960111

[B6] Evangelista L Cervino AR Ghiotto C Al-Nahhas A Rubello D Muzzio PC Tumor marker-guided PET in breast cancer patients-a recipe for a perfect wedding: a systematic literature review and meta-analysis Clin Nucl Med 2012 37 467 474 10.1097/RLU.0b013e31824850b0 22475896

[B7] Grassetto G Fornasiero A Otello D Bonciarelli G Rossi E Nashimben O Minicozzi AM Crepaldi G Pasini F Facci E Mandoliti G Marzola MC Al-Nahhas A Rubello D 18 F-FDG-PETCT in patients with breast cancer and rising CA15-3 with negative conventional imaging: a multicentre study Eur J Radiol 2011 80 828 833 10.1016/j.ejrad.2010.04.029 20547020

[B8] Suárez M Pérez-Castejón MJ Jiménez A Domper M Ruiz G Montz R Carreras JL Early diagnosis of recurrent breast cancer with FDG-PET in patients with progressive elevation of serum tumour markers Q J Nucl Med 2002 46 113 121 12114874

[B9] Mirabelli P Incoronato M Usefulness of traditional serum biomarkers for management of breast cancer patients Biomed Res Int 2013 doi:10.1155/2013/68564 10.1155/2013/685641 PMC3856124 24350285

[B10] Haug AR Schmidt GP Klingenstein A Heinemann V Stieber P Priebe M la Fougère C Becker C Hahn K Tiling R F-18-fluoro-2-deoxyglucose positron emission tomography/computed tomography in the follow-up of breast cancer with elevated levels of tumour markers J Comput Assist Tomogr 2007 31 629 634 10.1097/01.rct.0000284394.83696.42 17882045

[B11] Molina R Barak V van Dalen A Duffy MJ Einarsson R Gion M Goike H Lamerz R Nap M Sölétormos G Stieber P Tumor markers in breast cancer – European group on tumour markers recommendations Tumor Biol 2005 26 281 293 10.1159/000089260 16254457

[B12] European group on tumour marker. EGTM guideline for breast cancer Available from: http://www.egtm.eu/professionals/breast_cancer. Accessed 2 April 2013

[B13] Erdi YE Mawlawi O Larson SM Imbriaco M Yeung H Finn R Humm JL Segmentation of lung lesion volume by adaptive positron emission tomography image thresholding Cancer 1997 80 2505 2509 10.1002/(SICI)1097-0142(19971215)80:12+<2505::AID-CNCR24>3.0.CO;2-F 9406703

[B14] Evangelista L Baretta Z Vinante L Cervino AR Gregianin M Ghiotto C Saladini G Sotti G Tumour markers and FDG PETCT for prediction of disease relapse in patients with breast cancer Eur J Nucl Med Mol Imaging 2011 38 293 301 10.1007/s00259-010-1626-7 20882280

[B15] McMahon CJ Crowley V McCarroll N Dunne R Keogan MT Elevated tumour marker: an indication for imaging? Ann Clin Biochem 2010 47 4 327 330 10.1258/acb.2010.009235 20511377

[B16] Duffy MJ Evoy D McDermott EW CA15-3: uses and limitation as a biomarker for breast cancer Clin Chim Acta 2010 411 1869 1874 10.1016/j.cca.2010.08.039 20816948

[B17] Khatcheressian JL Hurley P Bantug E Esserman LJ Grunfeld E Halberg F Hantel A Henry NL Muss HB Smith TJ Vogel VG Wolff AC Somerfield MR Davidson NE Breast cancer follow-up and management after primary treatment: American Society of Clinical Oncology clinical practice guideline update J Clin Oncol 2013 31 7 961 965 10.1200/JCO.2012.45.9859 23129741

[B18] Sturgeon CM Duffy MJ Stenman UH Lilja H Brünner N Chan DW Babaian R Bast RC Jr Dowell B Esteva FJ Haglund C Harbeck N Hayes DF Holten-Andersen M Klee GG Lamerz R Looijenga LH Molina R Nielsen HJ Rittenhouse H Semjonow A Shih IM Sibley P Sölétormos G Stephan C Sokoll L Hoffman BR Diamandis EP National Academy of Clinical Biochemistry: National Academy of Clinical Biochemistry laboratory medicine practice guidelines for use of tumor markers in testicular, prostate, colorectal, breast, and ovarian cancers Clin Chem 2008 54 12 e11 e79 doi:10.1373/clinchem.2008.105601 10.1373/clinchem.2008.105601 19042984

[B19] Boellaard R O’Doherty MJ Weber WA Mottaghy FM Lonsdale MN Stroobants SG Oyen WJ Kotzerke J Hoekstra OS Pruim J Marsden PK Tatsch K Hoekstra CJ Visser EP Arends B Verzijlbergen FJ Zijlstra JM Comans EF Lammertsma AA Paans AM Willemsen AT Beyer T Bockisch A Schaefer-Prokop C Delbeke D Baum RP Chiti A Krause BJ FDG PET and PET/CT: EANM procedure guidelines for tumour PET imaging: version 1.0 Eur J Nucl Med Mol Imaging 2010 37 181 200 10.1007/s00259-009-1297-4 19915839 PMC2791475

[B20] Champion L Brain E Giraudet AL Le Stanc E Wartski M Edeline V Madar O Bellet D Pecking A Alberini JL Breast cancer recurrence diagnosis suspected on tumour marker rising: value of whole-body 18FDG-PET/CT imaging and impact on patient management Cancer 2011 117 1621 1629 10.1002/cncr.25727 21472709

[B21] Filippi V Malamitsi J Vlachou F Laspas F Georgiou E Prassopoulos V Andreou J The impact of FDG-PET/CT on the management of breast cancer patients with elevated tumor markers and negative or equivocal conventional imaging modalities Nucl Med Commun 2011 32 85 90 10.1097/MNM.0b013e328341c898 21127445

[B22] Evangelista L Baretta Z Vinante L Cervino AR Gregianin M Ghiotto C Bozza F Saladini G Could the serial determination of Ca15.3 serum improve the diagnostic accuracy of PET/CT? Results from small population with previous breast cancer Ann Nucl Med 2011 25 469 477 10.1007/s12149-011-0488-9 21476056

[B23] Katayama T Kubota K Machida Y Toriihara A Shibuya H Evaluation of sequential FDG-PET/CT for monitoring bone metastasis of breast cancer during therapy: correlation between morphological and metabolic changes with tumor markers Ann Nucl Med 2012 26 426 435 10.1007/s12149-012-0595-2 22477261

[B24] Sölétormos G Nielsen D Schiøler V Mouridsen H Dombernowsky P Monitoring different stages of breast cancer using tumour markers CA 15–3, CEA and TPA Eur J Cancer 2004 40 4 481 486 10.1016/j.ejca.2003.10.015 14962712

[B25] Laessig D Nagel D Heinemann V Untch M Kahlert S Bauerfeind I Stieber P Siggelkow W Rath W Buell U Zimny M FDG PET and tumour markers in the diagnosis of recurrent and metastatic breast cancer Eur J Nucl Med Mol Imaging 2004 31 Suppl 1 S118 S124 15146295 10.1007/s00259-004-1534-9

[B26] Mariani L Miceli R Michilin S Gion M Serial determination of CEA and CA 15.3 in breast cancer follow-up: an assessment of their diagnostic accuracy for the detection of tumour recurrences Biomarkers 2009 14 130 136 10.1080/13547500902770090 19330591

[B27] Nicolini A Carpi A Ferrari P Anselmi L Spinelli C Conte M Miccoli P The role of tumour markers in improving the accuracy of conventional chest X-ray and liver echography in the post-operative detection of thoracic and liver mestases from breast cancer Br J Cancer 2000 83 1412 1417 10.1054/bjoc.2000.1477 11076646 PMC2363419

